# Diagnostic Challenges in Follicular Cholangitis Mimicking Hilar Cholangiocarcinoma: A Case Report and Review of the Literature

**DOI:** 10.3390/medicina60091513

**Published:** 2024-09-17

**Authors:** Jungnam Lee, Seok Jeong, Don Haeng Lee, Suk Jin Choi, Woo Young Shin

**Affiliations:** 1 Department of Internal Medicine, Inha University Hospital, Inha University School of Medicine, Incheon 22332, Republic of Korea; jungnamlee@inha.ac.kr (J.L.); inos@inha.ac.kr (S.J.); ldh@inha.ac.kr (D.H.L.); 2 Department of Pathology, Inha University Hospital, Inha University School of Medicine, Incheon 22332, Republic of Korea; 204058@inha.ac.kr; 3 Department of Surgery, Inha University Hospital, Inha University School of Medicine, Incheon 22332, Republic of Korea

**Keywords:** follicular cholangitis, cholangiocarcinoma, diagnosis, case report

## Abstract

*Introduction*: Distinguishing benign from malignant biliary strictures remains challenging despite diagnostic advancements. Follicular cholangitis, a rare benign condition, presents with symptoms and imaging similar to malignancies like cholangiocarcinoma, often complicating diagnosis, particularly when tumor markers are elevated and imaging suggests metastasis. *Case presentation*: A 57-year-old woman with hypertension and diabetes was admitted with jaundice. Elevated bilirubin and liver enzymes alongside high carbohydrate antigen 19-9 (CA19-9) levels but normal carcinoembryonic antigen (CEA) were noted. Imaging showed thickening from the hilar duct to the proximal common bile duct, accompanied by suspected lymph node metastases. Comprehensive ERCP-guided biopsies found no malignancy. Surgical resection led to a diagnosis of follicular cholangitis. *Conclusions*: Follicular cholangitis’ long-term prognosis is elusive due to its rarity, and preoperative diagnosis is challenging. Increased awareness may improve diagnostic and treatment approaches, as this case adds to the disease’s understanding.

## 1. Introduction

Biliary strictures can arise from either benign or malignant diseases. In patients presenting with a biliary stricture and no visible mass on cross-sectional imaging, the estimated risk of malignancy is approximately 55% [1]. Benign biliary strictures may result from various causes, including hepatolithiasis, primary sclerosing cholangitis, and IgG4-related sclerosing cholangitis [2]. Despite remarkable advancements in diagnostic technologies, differentiating between benign biliary strictures and malignant diseases such as cholangiocarcinoma continues to pose a significant clinical challenge in real-world practice. This diagnostic complexity is further compounded by the overlapping clinical and imaging presentations of both malignant and benign conditions.

Follicular cholangitis, initially elucidated by Aoki et al. in 2003, has recently been recognized as a key differential diagnosis in cases of benign biliary strictures [3]. It presents with symptoms and imaging characteristics, such as segmental bile duct stricture and dilation, that closely resemble those of cholangiocarcinoma. Histopathologically, follicular cholangitis is characterized by localized fibrotic thickening of the bile duct wall and lymphocytic infiltration, leading to the formation of lymphoid follicles with germinal centers beneath the mucosal layer. Despite these distinctive features, it is prone to misdiagnosis as other autoimmune biliary disorders or cholangiocarcinoma. This report describes a case of follicular cholangitis that presented significant diagnostic challenges, including markedly elevated carbohydrate antigen 19-9 (CA19-9, 215.0 U/mL; reference: 0.0–34.0 U/mL) levels and accompanying regional lymph node metastasis observed in imaging studies, rendering it indistinguishable from malignancy. Initially suspected to be hilar cholangiocarcinoma with lymph node metastasis, the case was subsequently identified as follicular cholangitis during surgical exploration.

## 2. Case Presentation

**Figure 1:**
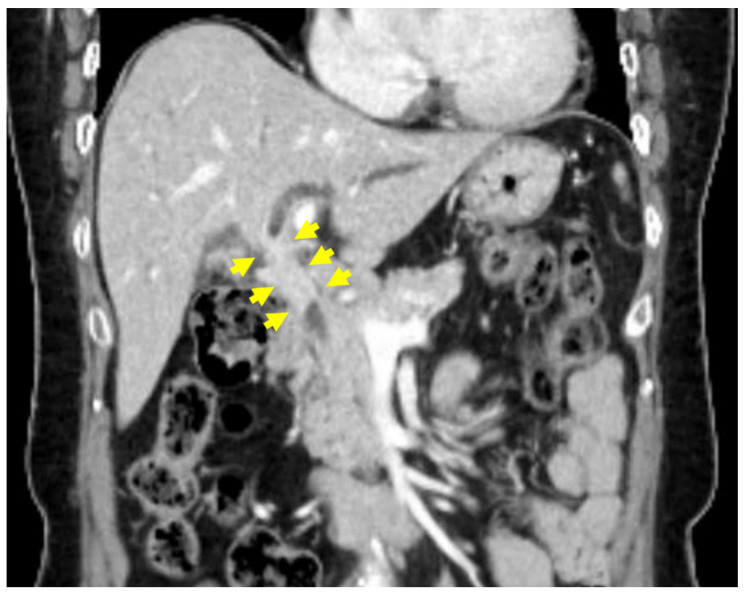
Biliary protocol CT, biliary CT showed a 47 mm thickening from the hilar to proximal common bile duct (yellow arrows).

**Figure 2:**
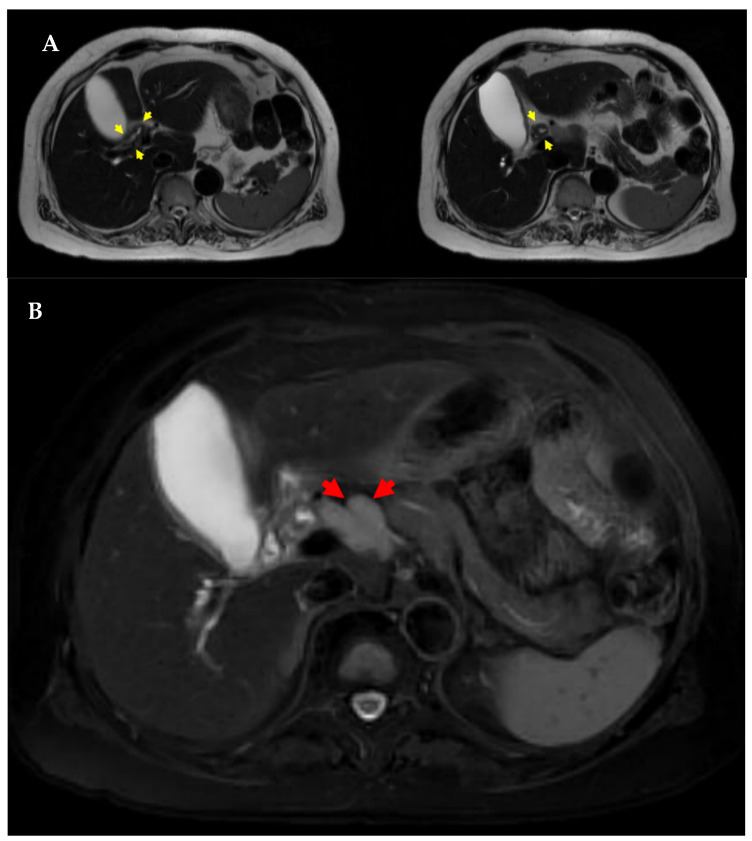
Liver protocol-MRI (**A**). Liver MRI indicated irregular thickening and narrowing at the hepatic duct bifurcation within the common hepatic duct (yellow arrows). (**B**). Enlarged lymph nodes over 1 cm were noted in the pericholedochal and portocaval regions (red arrows).

**Figure 3:**
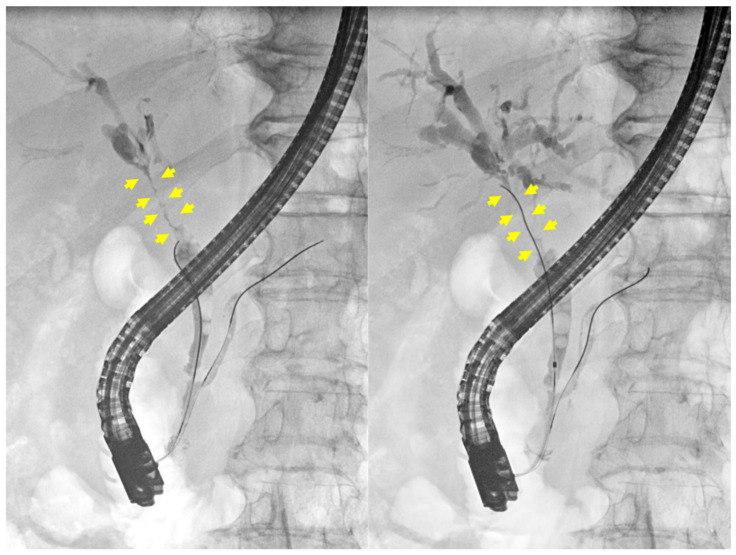
ERCP showed irregular narrowing in the hilar bile duct (yellow arrows) to the common bile duct and dilated intrahepatic ducts.

A 57-year-old female patient with a history of hypertension and type 2 diabetes mellitus was initially managed by a local physician before being referred to our hospital for further evaluation. She presented with jaundice, which had developed one week prior to her hospital visit. The patient did not report any additional symptoms aside from jaundice. Her medical history was unremarkable for biliary tract disease, previous abdominal surgery, autoimmune disorders, allergies, or abdominal trauma. Additionally, she had no documented history of excessive alcohol consumption or tobacco use. The patient exhibited a stable general condition and maintained a normal body mass index (25.6 kg/m^2^). Laboratory examinations revealed elevated levels of serum total bilirubin (10.43 mg/dL; reference: 0.00–1.20 mg/dL), direct bilirubin (9.70 mg/dL; reference: 0.00–0.30 mg/dL), aspartate aminotransferase (104 U/L; reference: 5–40 U/L), alanine aminotransferase (128 U/L; reference: 5–41 U/L), alkaline phosphatase (338 U/L; reference: 35–130 U/L), and γ-glutamyl transpeptidase (371 U/L; reference: 6–42 U/L). The serum CA19-9 (215.0 U/mL; reference: 0.0–34.0 U/mL) and IgG4 (119.5 mg/dL; reference: 3.9–86.4 mg/dL) levels were elevated, but carcinoembryonic antigen (CEA) levels remained within the normal range. Anti-mitochondrial antibody testing yielded a negative result. The biliary protocol computed tomography (CT) revealed a 47 mm segment of enhancing wall thickening in the region extending from the hilar duct to the proximal common bile duct (Figure 1). Additionally, there were suspicions of metastatic lymph nodes in the common hepatic area, hepatic duct confluence, and porta hepatis, along with a few indeterminate lymph nodes in the aortocaval and left paraaortic regions. The liver protocol MRI revealed irregular circumferential wall thickening with segmental narrowing at the hepatic duct bifurcation, specifically in the common hepatic duct (Figure 2A). Additionally, multiple enlarged lymph nodes were observed in the pericholedochal and portocaval spaces, each exceeding 1 cm in short axis measurement (Figure 2B). ERCP (endoscopic retrograde cholangiopancreatography) demonstrated irregular stenosis of the lumen within the hilar bile duct extending to the common bile duct, accompanied by a dilation of both intrahepatic ducts (Figure 3). Multiple biliary biopsies and brushing cytology procedures were performed on two separate occasions, but none of the specimens provided evidence of malignant disease. Immunostaining for IgG4 showed no significant infiltration of IgG4-positive plasma cells.

**Figure 4:**
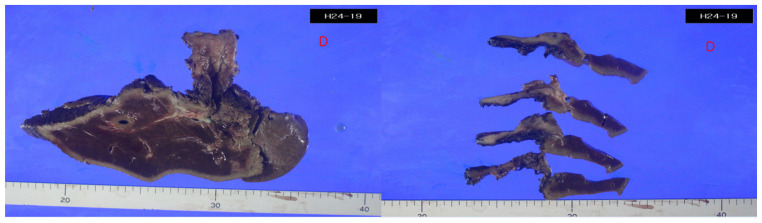
Resected specimen. The resected specimen shows diffuse thickening of the extrahepatic bile duct.

**Figure 5:**
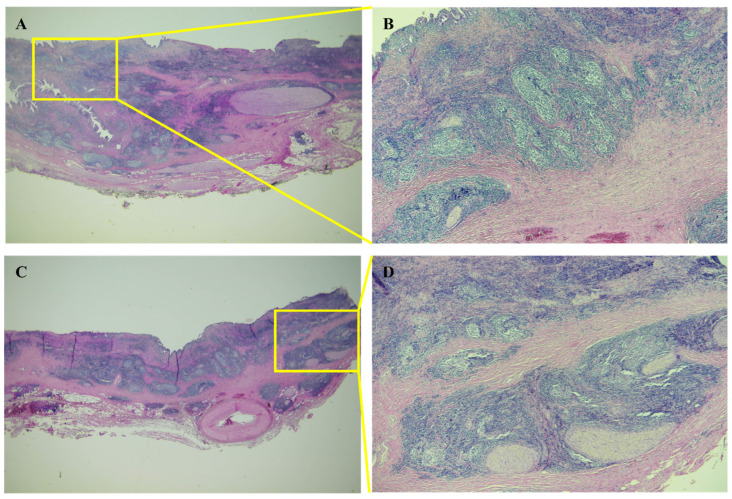
Microscopic findings of bile duct (hematoxylin & eosin). Hematoxylin and eosin-stained sections revealed diffuse thickening of the bile duct wall due to a transmural lymphoid infiltrate (**A**), the formation of hyperplastic lymphoid follicles (**B**), and extensive extension into the pericholedochal soft tissue (**C**,**D**). Follicular lymphoid hyperplasia, indicative of immune response activation.

**Figure 6:**
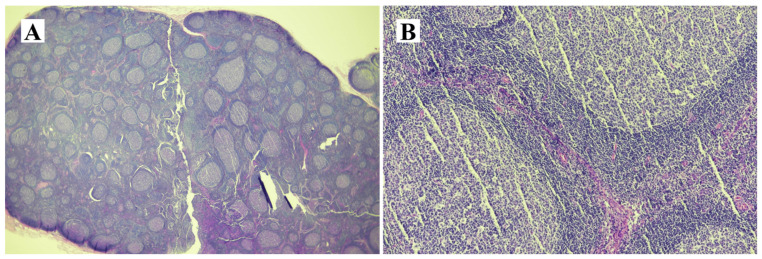
Microscopic findings of lymph node (hematoxylin & eosin). The enlarged lymph node also exhibited pronounced reactive follicular lymphoid hyperplasia (**A**,**B**).

**Figure 7:**
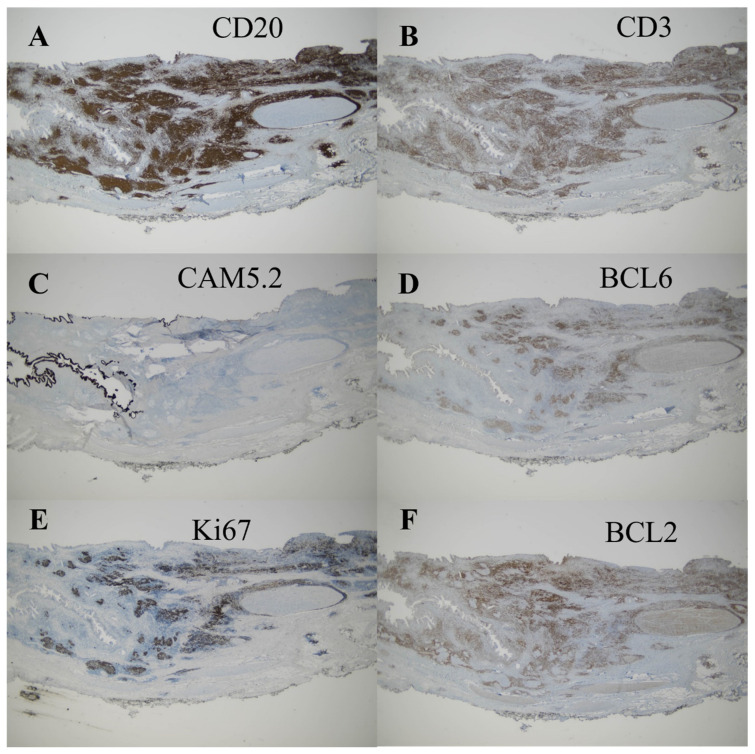
Immunohistochemistry (IHC) staining. The lymphoid follicles that are surrounded by mixed populations of CD20-immunoreactive B cells (**A**) and CD3-immunoreactive T cells (**B**) without forming lymphoepithelial lesions (**C**). The lymphoid follicles show immunophenotype of follicles (**D**,**E**) without aberrant expression of BCL2 (**F**).

Given the inability to definitively exclude hilar cholangiocarcinoma as a possibility in this case, a surgical approach was deemed appropriate and performed with the patient’s informed consent. The surgical procedures involved extended right hemihepatectomy and hepaticojejunostomy, along with regional lymph node dissection. The patient recovered well postoperatively and was discharged on the 16th day after surgery. In the operative field, the frozen biopsy from the left hepatic duct revealed chronic nonspecific inflammatory cell infiltration, characterized by nodular and focally diffused small lymphoid infiltration. The resected specimen exhibited diffusely thickened extrahepatic duct with enlarged lymph nodes (Figure 4). Upon microscopic examination, hematoxylin and eosin-stained sections showed diffuse thickening of the bile duct wall, with a dense transmural lymphoid infiltrate forming hyperplastic lymphoid follicles and extending into the adjacent pericholedochal soft tissue (Figure 5A–D). The enlarged lymph node also exhibited pronounced reactive follicular lymphoid hyperplasia (Figure 6A,B). Immunohistochemistry (IHC) staining revealed CD20 immunoreactivity in B cells and CD3 immunoreactivity in T cells surrounding the lymphoid follicles. The immunophenotypic profile of these follicles conforms to the standard follicular architecture and shows no atypical expression of BCL2. (Figure 7A–F). CD138, IgG, and IgG4-positive plasma cells were sparsely infiltrated with no significant presence. Kappa and lambda light chain reactions were not observed. Given these findings, the lesion was definitively diagnosed as follicular cholangitis. The patient has since been under outpatient follow-up care.

## 3. Discussion

Despite advances in diagnostic technology, accurately distinguishing between malignancy and benign conditions in biliary strictures remains challenging. Furthermore, even with improved endoscopic methods for obtaining tissue samples, up to 20% of patients initially diagnosed with cholangiocarcinoma are found to have benign conditions following surgical resection [4,5]. Benign bile duct stenosis includes various conditions such as iatrogenic biliary ductal injury, which is most frequently reported following hepatobiliary surgeries, with cholecystectomy and orthotopic liver transplantation being the leading causes [6]. Though less common, other causes of benign biliary stricture include post-cholangitis or pancreatitis-related biliary ductal inflammation, autoimmune cholangiopathies (immunoglobulin G4-mediated), biliary infections (including bacterial, parasitic, and human immunodeficiency virus), choledocholithiasis, Mirizzi syndrome, choledochal cysts, radiation-induced sclerosing cholangitis, biliary ductal ischemia, and trauma.

Follicular cholangitis is a rare condition distinct from other well-known benign biliary tract disorders. Since its initial description by Aoki et al. in 2003, only 12 cases have been documented in the literature. The literature review covered studies published up until 2023 and was conducted using the PubMed platform. Based on prior reports, including the present case, the median age of patients was 60 years, with an age range of 42 to 77 years (Table 1). The male-to-female ratio was 5:8, indicating a higher prevalence among female patients. Elevated total bilirubin was observed in seven cases but not observed in five cases, whereas gamma-glutamyl transpeptidase and alkaline phosphatase were elevated in all cases but one. None of the cases exhibited elevated serum CEA levels. Elevated CA19-9 levels were observed in only three reported cases. Additionally, there have been no previous instances in our experience where lymph node metastasis was suspected, or lymph node enlargement accompanied the diagnosis in imaging studies prior to diagnosis. This case we encountered suggests that even when lymph node enlargement is present, suggesting potential metastasis, and CA19-9 levels are markedly elevated, the possibility of a benign cause, particularly follicular cholangitis, should not be completely ruled out.

Localized bile duct stenosis has been consistently observed across reported cases. Follicular cholangitis predominantly affects the intrahepatic or hilar bile ducts, with only a single instance involving the distal bile duct. The factors contributing to the higher incidence of lesions in the proximal bile ducts compared to the distal biliary tract have not yet been fully elucidated. Preoperatively, the diagnoses were as follows: hilar cholangiocarcinoma in 9 cases, intrahepatic cholangiocarcinoma in 1 case, distal common bile duct cancer in 1 case, primary sclerosing cholangitis in 1 case, and hepatolithiasis in 1 case. Among the 13 cases we reviewed, malignancy was suspected in 11. In these cases, diagnostic imaging such as CT, magnetic resonance cholangiopancreatography (MRCP), and ERCP was performed, but they did not yield characteristic findings definitive for cholangiocarcinoma. Notably, the stricture segments were not multiple but presented as singular lesions, a feature that may help differentiate from primary sclerosing cholangitis. However, this still presents a challenge in distinguishing cholangiocarcinoma based solely on imaging. Particularly in this case, where findings suggestive of lymph node metastasis were present, accompanied by an elevation in serum CA 19-9, differentiating it from cholangiocarcinoma becomes even more challenging. Ultimately, in all 13 reported cases, surgery was performed, following which the diagnosis of follicular cholangitis was established postoperatively.

Furthermore, there have been various endeavors to enhance the diagnostic yield in the assessment of biliary strictures with undetermined etiology. Following a previous non-diagnostic ERCP, attempts have been made to improve diagnostic outcomes through the use of ERCP with cholangioscopy-guided biopsy sampling or EUS with fine needle aspiration (FNA) or fine needle biopsy (FNB) [15,16,17,18]. However, the widespread adoption of the ERCP with cholangioscopy-guided biopsy sampling approach is hindered by the cost and availability of the cholangioscopy system. Regarding the EUS with FNA or FNB techniques, there is a need to consider the significant risk of needle-tract seeding that may occur during the procedure in case of malignancy [19]. Artificial intelligence based on deep learning, a type of machine learning that can process complex patterns from raw data, has emerged as a promising tool for diagnosing biliary strictures [20]. Algorithms like convolutional neural networks have shown a high level of accuracy in distinguishing between benign and malignant strictures by analyzing endoscopic and imaging data [21]. However, further validation is required before artificial intelligence-based diagnostic methods can be implemented in clinical practice. Therefore, further research is warranted to enhance the diagnostic yield in cases of biliary strictures.

Follicular cholangitis can be considered a non-life-threatening disease. However, due to the very low prevalence of this disease, there are no reports yet on its long-term prognosis, nor has there been confirmation regarding the possibility of it being a premalignant lesion. Preoperative diagnosis of this disease remains challenging. However, with the increasing number of reported cases of follicular cholangitis, improvements in diagnostic accuracy and treatment strategies are anticipated.

## Figures and Tables

**Table 1:** Literature review of cases diagnosed with follicular cholangitis.

Case	Author /Region	Year	Age/Sex	Increase in Bilirubin or Liver Enzyme	CA19-9 Elevation	Location	Preoperative Diagnosis	CD3	CD20	Treatment	Prognosis
1	Aoki [3] /Japan	2003	57/F	+	−	Hilar	Hilar cholangiocarcinoma	+	+	Extended right hepatectomy	no data
2	Lee [7] /Korea	2005	61/M	+	no data	Hilar, right and left hepatic duct, cystic duct, gallbladder neck	Hilar cholangiocarcinoma	no data	no data	Extrahepatic bile duct and part of the right hepatic duct resection	no data
3, 4	Fujita [8] /Japan	2010	47/M	+	−	Hilar, right hepatic duct	Hilar cholangiocarcinoma	+	+	Extended right hepatectomy	10 m alive
			44/F	−	−	Hilar	Hilar cholangiocarcinoma	+	+	Extended left hepatectomy	24 m dead
5, 6, 7	Zen [9] /Japan	2012	73/F	+	+	Right hepatic duct	Hilar cholangiocarcinoma	+	+	Right hepatectomy	no data
			70/M	+	+	Left perihilar duct	Hilar cholangiocarcinoma	+	+	Left hepatectomy	no data
			42/F	+	−	Hilar	Primary sclerosing cholangitis	+	+	Liver transplantation	no data
8	Fujii [10] /Japan	2014	60/F	+	−	Hilar, right and left hepatic duct	Hilar cholangiocarcinoma	no data	no data	Left trisegmentectomy	24 m alive
9	Saito [11] /Japan	2016	69/F	−	−	B3	Hepatolithiasis	no data	+	Left hepatectomy	12 m alive
10	Chang [12] /Japan	2019	60/M	+	−	Right hepatic duct posterior branch	Hilar cholangiocarcinoma	+	+	Right hepatectomy and caudate lobectomy	18 m alive
11	Kosone [13] /Japan	2020	70/M	+	−	Hilar	Hilar cholangiocarcinoma	no data	no data	Hilar cholangiocarcinoma	30 m alive
12	Koneri [14] /Japan	2023	77/F	+	−	Distal bile duct	Distal bile duct cancer	+	+	Subtotal stomach-preserving pancreaticoduodenectomy	42 m alive
13	Lee (our case) /Korea	2023	57/F	+	+	Hilar	Hilar cholangiocarcinoma			Subtotal stomach-preserving pancreaticoduodenectomy	3 m alive

## Data Availability

The data presented in this study are available on request from the corresponding author.

## Abbreviations

CA 19-9, carbohydrate antigen 19-9; CEA, CT, computed tomography; MRI, magnetic resonance imaging; ERCP, endoscopic retrograde cholangiopancreatography; IHC, immunohistochemistry; MRCP, magnetic resonance cholangiopancreatography; EUS, endoscopic ultrasound; FNA, fine needle aspiration; FNB, fine needle biopsy
